# Nimbolide inhibits pancreatic cancer growth and metastasis through ROS-mediated apoptosis and inhibition of epithelial-to-mesenchymal transition

**DOI:** 10.1038/srep19819

**Published:** 2016-01-25

**Authors:** Ramadevi Subramani, Elizabeth Gonzalez, Arunkumar Arumugam, Sushmita Nandy, Viviana Gonzalez, Joshua Medel, Fernando Camacho, Andrew Ortega, Sandrine Bonkoungou, Mahesh Narayan, Alok kumar Dwivedi, Rajkumar Lakshmanaswamy

**Affiliations:** 1 Center of Excellence in Cancer Research, Department of Biomedical Sciences MSB1, Texas Tech University Health Sciences Center, Paul L. Foster School of Medicine, El Paso, Texas-79905; 2 Graduate School of Biomedical Sciences, Texas Tech University Health Sciences Center, El Paso, Texas-79905; 3 The University of Texas at El Paso, El Paso, TX 79968; 4 Biostatistics and Epidemiology Consulting Laboratory, Texas Tech University Health Sciences Center, El Paso, Texas-79905.

## Abstract

The mortality and morbidity rates of pancreatic cancer are high because of its extremely invasive and metastatic nature. Its lack of symptoms, late diagnosis and chemo–resistance and the ineffective treatment modalities warrant the development of new chemo–therapeutic agents for pancreatic cancer. Agents from medicinal plants have demonstrated therapeutic benefits in various human cancers. Nimbolide, an active molecule isolated from *Azadirachta indica*, has been reported to exhibit several medicinal properties. This study assessed the anticancer properties of nimbolide against pancreatic cancer. Our data reveal that nimbolide induces excessive generation of reactive oxygen species (ROS), thereby regulating both apoptosis and autophagy in pancreatic cancer cells. Experiments with the autophagy inhibitors 3-methyladenine and chloroquine diphosphate salt and the apoptosis inhibitor z-VAD-fmk demonstrated that nimbolide-mediated ROS generation inhibited proliferation (through reduced PI3K/AKT/mTOR and ERK signaling) and metastasis (through decreased EMT, invasion, migration and colony forming abilities) via mitochondrial-mediated apoptotic cell death but not via autophagy. *In vivo* experiments also demonstrated that nimbolide was effective in inhibiting pancreatic cancer growth and metastasis. Overall, our data suggest that nimbolide can serve as a potential chemo–therapeutic agent for pancreatic cancer.

Early detection of pancreatic cancer is critical because of its severe metastatic and invasive nature^1^. It is the 4^th^ leading cause of cancer-related death in the US and is projected to become the 2^nd^ leading cause of death by the year 2020^2^,^3^. Pancreatic cancer is the only major cancer with a 5-year survival rate of less than 6%, indicating that it is one of the deadliest cancers^4^. Since a high proportion of cancers are diagnosed at late-stages, only 10–20% of pancreatic cancer patients are suitable for surgical resection of the cancer^5^,^6^. With no effective treatments currently available, there is an immediate need for the development of novel therapeutic agents.

Several plant-derived compounds have been widely used as therapeutic agents against cancers^7^,^8^,^9^,^10^. Nimbolide, a phytochemical isolated from the leaves and flowers of the neem tree (*Azadirachta indica*), has been used as a traditional folk medicine for a variety of human illnesses^11^,^12^. Various phytochemicals have been shown to exhibit their anticancer properties by altering the levels of reactive oxygen species^13^,^14^. Reactive oxygen species (ROS) are byproducts of oxygen metabolism that occur under cellular oxidative stress conditions and play a vital role in the maintenance of homeostasis via redox pathways. Based on the levels of its generation, ROS can either have beneficial or adverse effects on cells^15^,^16^. Pancreatic cancer cells generate moderate levels of ROS to aid in their proliferation, migration, and metastasis. Most pancreatic cancers are highly resistant to apoptosis and chemo–therapeutic drugs partially due to this moderate ROS generation. Conversely, excessive levels of ROS promote pancreatic cancer cell death by creating a hostile environment^17^,^18^,^19^.

In the present study, we examined the anticancer effect of nimbolide on pancreatic cancer. Our data demonstrates that nimbolide-induced excessive ROS generation is the main cause of inhibition of pancreatic cancer growth and metastasis. Furthermore, we also demonstrate that ROS inhibits pancreatic cancer growth mainly through apoptosis and not through autophagy. Actually, our data indicates that the increased autophagy in response to nimbolide treatment is a defense mechanism that promotes cell survival. In conclusion, nimbolide is a promising anticancer agent that is highly effective against pancreatic cancer.

## Results

### Nimbolide inhibits the proliferation of human pancreatic ductal adenocarcinoma cells

**Figure 1:**
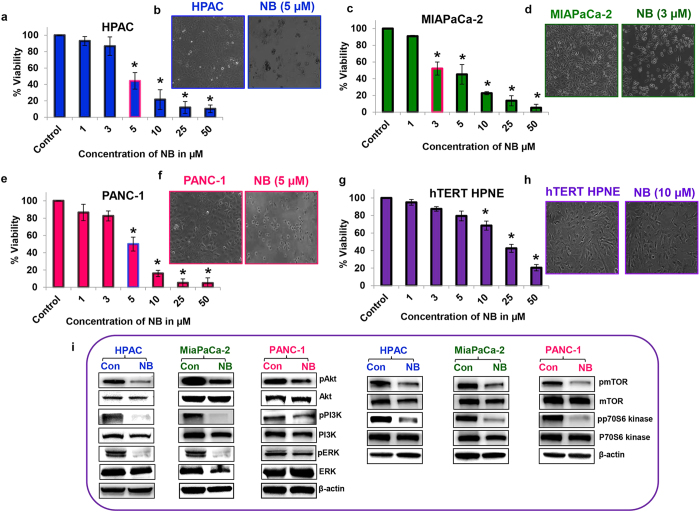
Anti–proliferative effect of nimbolide on non-malignant and malignant pancreatic cell lines. HPAC, MIAPaCa-2, PANC-1, and hTERT HPNE cells were treated with different concentrations of nimbolide (1–50 μM) for 24 h, and cell viability was assessed using the MTS assay (**a**,**c**,**d**,**g**). Each bar represent the mean±standard error of the mean (SEM) of three separate experiments, *p < 0.05. Morphology comparison of control and cells treated with the respective IC_50_ concentration of nimbolide (5 μM, 3 μM, and 5 μM for HPAC, MIAPaCa-2, PANC-1 cells, respectively) viewed under a light microscope at 10X magnification (**b**,**d**,**f**,**h**). Immunoblot analysis of cell proliferation signaling molecules in control and nimbolide-treated PDAC cell lines (**i**). Repeated measures analysis of variance and Dunnett post hoc test was performed to determine statistical significance.

Exponentially growing pancreatic cancer cells were treated with various concentrations of nimbolide, ranging from 1–50 μM, for 24 h. Nimbolide significantly inhibited the viability of the pancreatic cancer cell lines (HPAC, MIAPaCa-2, and PANC-1) in a dose-dependent manner (Fig. 1a,c,e). The cell viability data demonstrate that nimbolide induced more than 50% cell death at a concentration of 5 μM in HPAC and PANC-1 cells and at 3 μM in MIAPaCa-2 cells. However, 3 or 5 μM of nimbolide treatment did not significantly change the cell viability of the normal pancreatic cells (hTERT HPNE) compared with the untreated normal pancreatic cells (Fig. 1g). In fact, even at higher doses (10 μM), nimbolide was not effective in inducing cell death in normal pancreatic cells, which indicates a preferential effect of nimbolide on pancreatic cancer cells. Microscopic visualization of HPAC, MIAPaCa-2 and PANC-1 cells treated with the respective IC_50_ concentrations of nimbolide (5, 3 and 5 μM, respectively) for 24 h showed a dramatic change in the number of live cells and a significant alteration in cell morphology (Fig. 1b,d,f). In contrast, microscopic visualization of normal pancreatic cells even after 10 μM nimbolide treatment showed no significant alteration in the cell number or cell morphology (Fig. 1h). These results indicate that the selected optimum doses of nimbolide have a significant anticancer effect with minimal adverse effects on normal cells. The IC_50_ concentrations of nimbolide for each cell line were used for further experimental analysis (5 μM for HPAC and PANC-1 cells and 3 μM for MIAPaCa-2 cells). Analysis of key molecules involved in the induction of proliferation by immunoblots revealed that nimbolide treatment reduced the expression levels of the phosphorylated forms of AKT, PI3K, ERK, mTOR and p70S6 kinase in the pancreatic cancer cell lines;. The total protein levels of AKT, PI3K, ERK, mTOR and P70S6 kinase were similar in both control and nimbolide-treated HPAC and PANC-1 cells (Fig. 1i). Interestingly, a small reduction in the expression of total AKT and ERK was observed in nimbolide-treated MIAPaCa-2 cells. Blots against β-actin were performed as a control for equal protein loading.

### Nimbolide suppresses migration, invasion, EMT and anchorage-independent growth of pancreatic cancer cells

**Figure 2:**
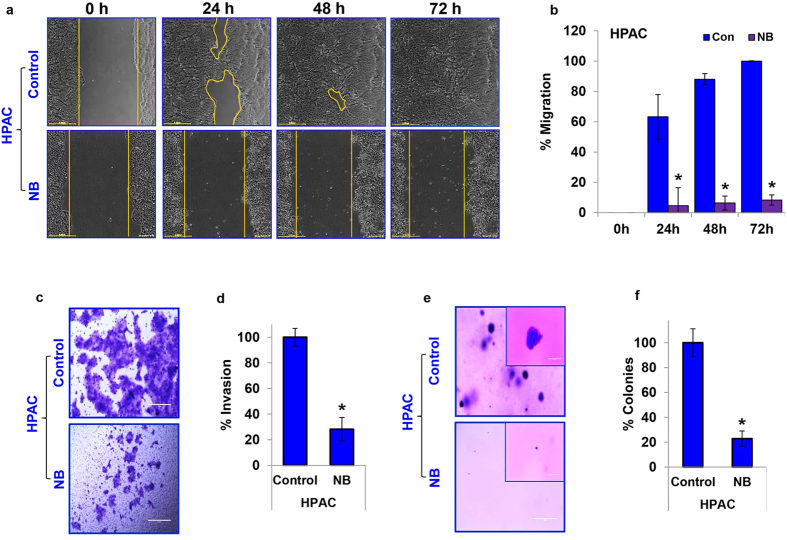
Nimbolide inhibits pancreatic cancer cell migratory, invasive, EMT and anchorage independent growth potential. A scratch assay in control HPAC cells and 5 μM nimbolide-treated HPAC cells was performed using an automated Nikon Biostation CT at 2-h intervals for up to 72 h (**a**). Quantitative analysis of migration in HPAC cells after nimbolide treatment was calculated with NIS-Element AR software (**b**). Invasiveness of PDAC cells was observed using a Matrigel invasion assay after 5 μM nimbolide treatment (**c**,**d**). A colony-formation assay performed with HPAC cells cultured with or without 5 μM nimbolide treatment (**e**). The percentage colonies were calculated with HPAC control cells serving as the baseline (**f**). Each bar represents the mean±SEM of three separate experiments, *p < 0.05. Paired-t test was performed to determine statistical significance.

Migration and invasion are the initial and critical events in metastasis and enhance the ability of a cancer cell to enter and exit the circulation to reach distant organs. The effect of nimbolide on pancreatic cancer cell migration was studied using the scratch assay. The migratory capability of HPAC cells treated with nimbolide was remarkably decreased, as evidenced by the fact that nimbolide-treated HPAC cells did not reform the monolayer (Fig. 2a,b) even at 72 h, while control cells reformed a complete monolayer within 48 h. Similarly, nimbolide was also effective in suppressing the migratory abilities of MIAPaCa-2 (Supplementary Fig. S1a,b) and PANC-1 cells (Supplementary Fig. S2a,b).

The interaction and communication between cancer cells and the microenvironment controls cancer progression. It is well established that increased invasion and anchorage-independent growth potentials favor metastasis. Matrigel-coated Boyden chambers were used to investigate the effect of nimbolide on the pancreatic cancer cells invasive capacity. Nimbolide significantly inhibited HPAC cell invasion by more than 70% compared with vehicle-treated HPAC cells (Fig. 2c,d). A similar effect of nimbolide treatment was also observed in the other two pancreatic cancer cell lines (MIAPaCa-2 cells; ~80%; Supplementary Fig. S1c,d; and PANC-1 cells; ~65%; Supplementary Fig. S2c,d). Furthermore, the soft agar colony-formation assay revealed the inhibitory effect of nimbolide on anchorage-independent growth potential of pancreatic cancer cells. Nimbolide drastically reduced the size and number of the colonies of HPAC cells by ~80% (Fig. 2e,f). Nimbolide treatment produced the same effect in MIAPaCa-2 cells (Supplementary Fig. S1e, f) and PANC-1 cells (Supplementary Fig. S2e, f), as their ability to form colonies were also decreased significantly. These results indicate that nimbolide effectively inhibits the migratory, invasive and colony-forming capabilities of pancreatic cancer cells.

**Figure 3:**
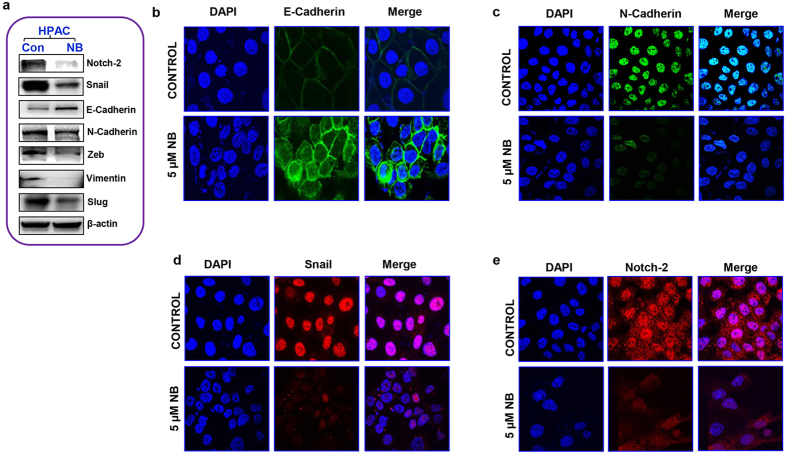
Nimbolide inhibits EMT in pancreatic cancer cells. Immunoblotting analysis of EMT marker expression levels in response to 5 μM nimbolide administration (**a**). Inhibition of EMT was recorded through immunofluorescence after both control and 5 μM nimbolide-treated HPAC cells were fixed and stained with anti– E-Cadherin (**b**), N-Cadherin (**c**), Snail (**d**) and Notch-2 (**e**) antibody visualized through fluorescence confocal microscopy with 100x magnification.

EMT is considered essential for cancer cells to invade, migrate and establish a metastatic colony. The expression of “EMT master genes” was analyzed to determine whether nimbolide inhibited pancreatic cancer cell migration and invasion by altering the EMT process. Nimbolide treatment decreased the expression of Notch-2, N-cadherin, vimentin and a family of transcription factors (Snail, Slug and Zeb). Additionally, nimbolide treatment also increased the expression of E-cadherin in HPAC cells (Fig. 3a). The same trend of EMT marker expression was also observed in the other two pancreatic cancer cell lines, indicating that nimbolide effectively inhibited EMT (Supplementary Fig. S1g and S2g). Immunofluorescence analysis for E-cadherin, N-cadherin, snail and Notch-2 in HPAC cells further confirmed the inhibition of EMT by nimbolide (Fig. 3b–e). Overall, these data clearly demonstrate that nimbolide modulates the migration, invasion and anchorage-independent growth of pancreatic cancer cells by inhibiting the EMT process.

### Nimbolide treatment induces reactive oxygen species generation, autophagy and apoptosis

**Figure 4:**
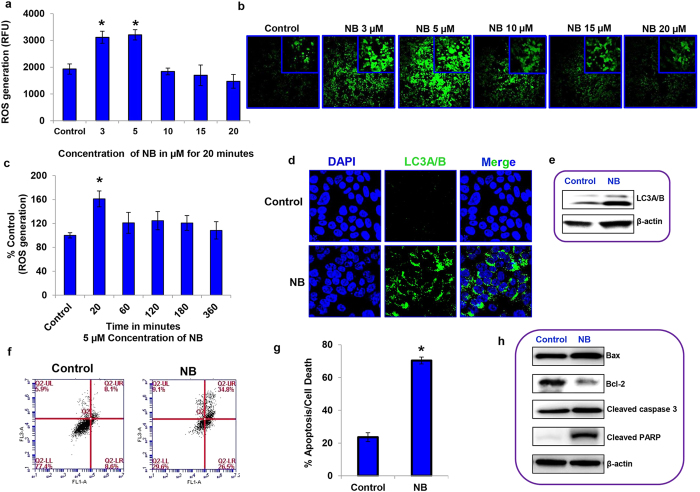
Nimbolide treatment induces ROS generation, autophagy and apoptosis in the HPAC pancreatic cancer cell line. Mitochondrial ROS levels in HPAC cells in response to different doses of nimbolide treatment (3, 5, 10, 15, and 20 μM) for 20 min using a fluorescence plate reader (**a**) and fluorescence confocal microscopy in 10X and 40X magnifications (**b**). Time-course analysis of ROS generation (**c**). Induction of autophagy was recorded through immunofluorescence after both control and 5 μM nimbolide-treated HPAC cells were fixed and stained with anti–LC3A/B antibody visualized through fluorescence confocal microscopy (**d**) and LC3A/B protein expression using immunoblotting analysis (**e**). A significant increase in the percentage of apoptosis after 5 μM nimbolide treatment was determined using an Annexin V-FITC Apoptosis Detection Kit I (**f**,**g**). The protein levels of Bax, Bcl-2, Caspase 3, and cleaved PARP confirmed the induction of apoptosis (**h**). Each bar represents the mean±SEM of three separate experiments, *p < 0.05. Repeated measures analysis of variance and Dunnett post hoc test was performed to determine statistical significance.

Excessive ROS production is associated with the induction of cell death and differentiation^20^. To investigate the effect of nimbolide on ROS generation, HPAC cells were exposed to 3–20 μM nimbolide for 20 minutes. Interestingly, in the dose-response analysis, an increased amount of ROS was generated at lower concentrations of nimbolide than at higher concentrations (Fig. 4a). Consistent with our previous results that were determined using a microplate reader, fluorescence analysis using confocal microscopy also demonstrated that 5 μM nimbolide induced maximum ROS production (Fig. 4b) at 20 min. A time-course experiment using 5 μM nimbolide demonstrated that ROS generation occurs early and remains stable up to 6 h (Fig. 4c). Similar effects of nimbolide on ROS generation and stability were also observed in the other two pancreatic cancer cells (Supplementary Fig. S3a–c and Supplementary Fig. S4a–c). These data indicate that the ROS generation is a rapid and an early event of nimbolide-induced anticancer effects. Furthermore, the dose-response experiment indicates that an optimum level of nimbolide is required to induce maximum ROS levels, as higher doses were ineffective at inducing ROS because of their strong cytotoxic effect.

Next, the influence of nimbolide on autophagy and apoptosis, the two major types of programmed cell death mechanisms, was investigated. Nimbolide stimulated autophagy, which was confirmed by increased punctate cytosolic LC3A/B fluorescence intensity (a marker of autophagy) based on confocal microscopy in all 3 PDAC cells (Fig. 4d, Supplementary Fig. S3d and S4d). Immunoblot assays also revealed the elevated expression levels of LC3A/B after nimbolide exposure in the pancreatic cancer cells (Fig. 4e, Supplementary Fig. S3e and S4e). To better understand the effect of nimbolide on apoptosis, flow cytometric analysis was performed using an annexin V–FITC apoptosis detection kit. Nimbolide treatment showed a pronounced induction of apoptosis/cell death at 24 h (70.4% in HPAC, 52.6% in MIAPaCa-2 and 31.1% in PANC-1; Fig. 4f, g, Supplementary Fig. S3f, g and Supplementary Fig. S4f, g). Molecular changes associated with nimbolide-induced apoptotic cell death were also determined using immuno blots. Most chemo–therapeutic agents induce apoptosis via an intrinsic mitochondrial-mediated pathway rather than the death receptor pathway^21^. Increased expression of pro-apoptotic Bax and cleaved/active forms of the executioners caspase-3 and PARP were recorded in HPAC cells after nimbolide treatment (Fig. 4h). We also found that the anti–apoptotic molecule Bcl-2 was down regulated upon nimbolide exposure (Fig. 4h). Based on our consistent results with two other cell lines, MIAPaCa-2 (Supplementary Fig. S3h) and PANC-1 (Supplementary Fig. S4h), it is evident that nimbolide induces apoptosis through the intrinsic mitochondrial-mediated pathway.

### Nimbolide-induced ROS regulate autophagy and apoptosis in PDAC cells

**Figure 5:**
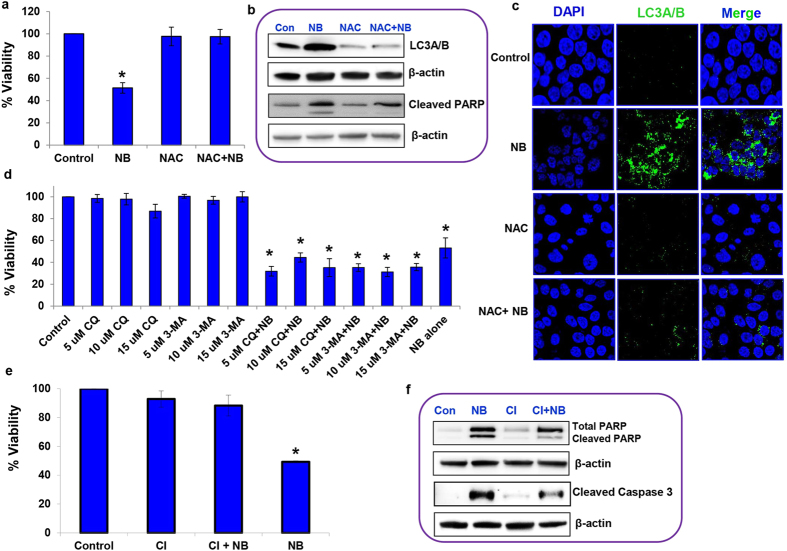
Induction of autophagy and apoptosis by nimbolide occurs through ROS generation. The cell viability of HPAC cells was assessed after treatment with nimbolide (5 μM) or NAC (5 mM) alone or in combination for 24 h using the MTS assay (**a**). The expression levels of LC3A/B and cleaved PARP were evaluated based on immunoblotting (**b**). The regulatory role of ROS on autophagy was further confirmed with immunostaining of LC3A/B (**c**). The autophagy inhibitor experiment was conducted with pretreatment of early- and late-stage autophagic inhibitors CQ and 3-MA, respectively, in the presence and absence of nimbolide (5 μM) to test the cell viability via the MTS assay (**d**). The caspase inhibitor, z-VAD-fmk (10 μM) (denoted CI) with and without nimbolide (5 μM) was used to determine the cell viability of HPAC cells (**e**). ROS-mediated apoptotic cell death was observed by analyzing cleaved PARP and cleaved Caspase 3 levels using immunoblotting (**f**). Each bar represents the mean±SEM of three separate experiments, *p < 0.05. Repeated measures analysis of variance and Dunnett post hoc test was performed to determine statistical significance.

HPAC cells were pre-treated with 5 mM of the ROS inhibitor N-acetyl-L-cysteine (NAC). After one hour, the cells were treated with 5 μM nimbolide to determine the effect of ROS on cell viability. Pre-treatment with the ROS inhibitor significantly blocked nimbolide-induced cell death (Fig. 5a). Furthermore, results from the MIAPaCa-2 (Supplementary Fig. S5a) and PANC-1 (Supplementary Fig. S6a) cells also confirmed that nimbolide-induced pancreatic cancer cell death through excessive ROS generation.

The regulatory role of ROS on autophagy and apoptosis was characterized by monitoring the changes in LC3A/B and PARP levels. Immunoblotting analysis demonstrated increased levels of LC3A/B in the presence of nimbolide compared with control, NAC alone or NAC with nimbolide combination groups (Fig. 5b, Supplementary Fig. S5b and S6b). In addition, using immunofluorescence, we examined the localization of LC3A/B in response to the above-mentioned treatments. Targeting ROS inhibited the nimbolide-induced cytosolic LC3A/B punctate changes, indicating that autophagy induced by nimbolide was through ROS (Fig. 5c, Supplementary Fig. S5c and S6c). Next, we determined the effect of ROS on apoptosis. ROS blockade decreased nimbolide-induced apoptosis, which was evident from the significantly reduced expression of cleaved PARP. NAC alone treatment failed to induce apoptosis compared with nimbolide treated cells (Fig. 5b, Supplementary Fig. S5b and S6b). These findings suggest that nimbolide-induced ROS regulates both programmed type I (apoptosis) and II (autophagy) cell-death mechanisms.

### Nimbolide-induced autophagy did not lead to cell death

Autophagy has been shown to positively or negatively influence pancreatic cancer growth^22^,^23^,^24^. Here, it was important to understand whether nimbolide-induced autophagy is cytoprotective or cytotoxic. To test the role of autophagy, HPAC cells were exposed to both early- and late-stage autophagy inhibitors, 3-methyladenine (3-MA) and chloroquine diphosphate salt (CQ), respectively, in the presence or absence of nimbolide. HPAC cells exposed to various concentrations of 3-MA and CQ (5–15 μM) exhibited minimal cytotoxicity (<14%) at 24 h. Our data demonstrate that inhibition of autophagy did not alter nimbolide-induced cell death (Fig. 5d; Supplementary Fig. S5d and S6d). The increased autophagy observed in pancreatic cancer cells is actually an adaptive stress response to extend cell viability rather than initiate autophagic cell death.

### Oxidative stress induces apoptosis-dependent cell death

To ascertain whether nimbolide-induced apoptosis is the main mechanism of overall cell death of pancreatic cancer, PDAC cells were exposed to a pan caspase inhibitor (z-VAD-fmk) either alone or in combination with nimbolide for 24 h. The inhibition of apoptosis by z-VAD-fmk significantly rescued nimbolide-induced cell death resulting in ~88.3% viable HPAC cells (Fig. 5e). Similarly, in MIAPaCa-2 and PANC-1 cells, inhibition of apoptosis by z-VAD-fmk (denoted as CI) also rescued nimbolide-induced cell death and eventually increased the percentage of viable cells by 74.4% and 77.1%, respectively (Supplementary Fig. S5f and S6f). These data suggest that nimbolide-induced cell death occurs mainly via apoptosis. z-VAD-fmk treatment also decreased the expression levels of nimbolide-induced cleaved PARP and cleaved Caspase 3 (Fig. 5f, Supplementary Fig. S5f and S6f). Experiments using the pan–caspase inhibitor confirmed that nimbolide-induced ROS-mediated apoptotic cell death in PDAC cells.

### Effect of nimbolide on tumor growth, autophagy, apoptosis, EMT and metastasis in a pancreatic cancer xenograft model

**Figure 6:**
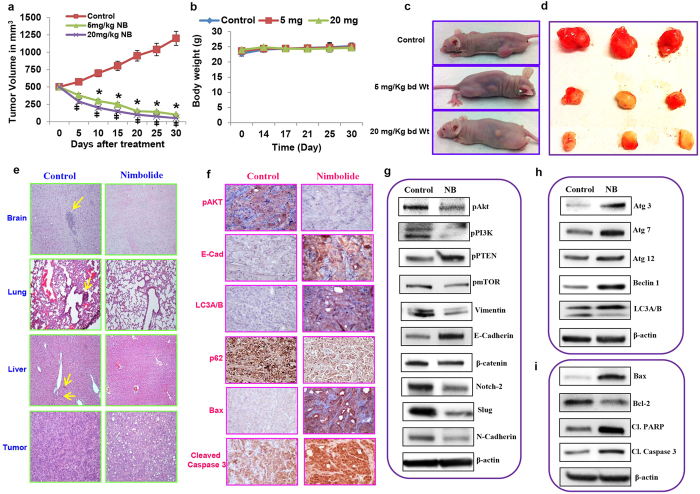
Effect of nimbolide on tumor growth, autophagy, apoptosis, EMT and metastasis in the HPAC xenograft model. The tumor volume of each group (control, 5 mg, and 20 mg nimbolide/Kg body weight) was recorded twice weekly (**a**). The unaltered body weight of the mice throughout this study of nimbolide treatment demonstrated the non-toxic effect of the highest concentration tested (20 mg/kg body weight) (**b**). Each bar represents the mean±SEM of three separate experiments, *p < 0.05. The mice were treated with different doses of nimbolide (**c**). Excised tumor xenografts (**d**). H&E staining of tumor sections demonstrating the inhibition of micrometastasis in the brain, lung and liver by nimbolide; tumor sections with reduced cell density confirmed the anti–tumor activity of nimbolide (**e**). Reduced pAKT expression and increased E-cadherin, LCA/B, p62, Bax, and cleaved Caspase 3 expression were observed in the nimbolide-treated mice, by using IHC staining (**f**). Immunoblotting was conducted on xenograft tissue samples to analyze the proliferation, EMT, autophagy, and apoptotic markers after nimbolide treatment (**g**–**i**). Repeated measures analysis of variance and Dunnett post hoc test was performed to determine statistical significance.

To extend our *in vitro* findings, we performed xenograft studies using HPAC cells. Three weeks after subcutaneous implantation of HPAC cells into nude mice, similar baseline tumor volumes were recorded in the mice. The mice bearing tumors (500 mm^3^) were randomly divided into three groups (vehicle control, 5 mg/kg body weight nimbolide and 20 mg/kg body weight nimbolide). Vehicle (DMSO) or nimbolide was administered intraperitoneally twice a week for 30 days. Nimbolide treatment dramatically decreased the tumor volume of HPAC xenografts compared with vehicle treatment (Fig. 6a). Both 5 and 20 mg/kg body weight dosage of nimbolide exhibited almost a similar growth inhibitory effect on pancreatic cancers without causing adverse effects in terms of body weight (Fig. 6b–d). Hematoxylin and eosin (H&E)-stained images of vehicle-treated control mice tissues exhibited micrometastases in the brain, lung and liver. Additionally, the tumor tissues from control mice were found to be highly cellular compared with the tissues from the nimbolide-treated mice. The mice treated with nimbolide showed no toxicity or metastasis in the brain, lung or liver, suggesting that nimbolide is a safe, natural therapeutic for PDAC (Fig. 6e). Because the anti–tumor effect of nimbolide was similar in mice treated with 5 or 20 mg/kg body weight dosage, we chose to use the 5 mg/kg body weight dose of nimbolide for all subsequent studies.

Next, we examined the expression levels of key proliferative, EMT, autophagy and apoptosis markers in tumor tissues derived from the control and nimbolide-treated mice. Nimbolide treatment resulted in significant down regulation of AKT phosphorylation compared with the control tumor group, as measured using IHC. Nimbolide inhibited the process of EMT by increasing the levels of epithelial marker E-cadherin. The autophagy regulatory molecule LC3A/B was also concurrently increased in nimbolide-treated mice resembling the *in vitro* observation. Sequestosome-1/p62 recognize and bind with toxic cellular waste during autophagy and its expression decreases as autophagy is induced^25^,^26^. Nimbolide treatment resulted in decreased expression of p62 indicating the induction of autophagy. Nimbolide induced stress increased autophagy at a higher frequency as a defensive mechanism for cell survival. Nimbolide induced the expression of the cleaved Caspase 3 and pro-apoptotic regulator Bax, which has a direct role in mitochondrial outer membrane permeabilization to release cytochrome-C leading to the activation of caspases, key enzymatic mediators of apoptosis (Fig. 6f). Overall, these results demonstrate that nimbolide inhibits pancreatic cancer cell proliferation and metastasis by inducing apoptosis.

Immunoblot analysis essentially confirmed the data obtained by immunohistochemical analysis to further emphasize the therapeutic role of nimbolide in pancreatic cancer. Similar to the *in vitro* findings, the expression levels of the phosphorylated forms of AKT, PI3K, and mTOR were suppressed in the nimbolide-treated group. In addition, the active form of PTEN was highly increased in the nimbolide-treated mice versus the tumor-bearing control mice (Fig. 5g). Nimbolide treatment decreased the expression of mesenchymal markers, such as vimentin, β-catenin, notch-2, slug, and N-cadherin, while it increased the expression of the epithelial marker E-cadherin (Fig. 5g). Nimbolide-induced autophagy correlated with the significant upregulation of Atg 3, 7, 12, Beclin and LC3A/B expression in pancreatic xenograft models (Fig. 5h). Pro-and anti–apoptotic molecules Bax and Bcl-2, respectively, are vital players in the activation of intrinsic mitochondrial-mediated apoptosis. In addition, the activation of caspases and the cleavage of its substrate poly (ADP-ribose) polymerase (PARP) are the characteristic hallmarks of apoptosis^27^. Increased expression of pro-apoptotic proteins Bax, cleaved caspase-3, and cleaved PARP; in addition, decreased expression of anti–apoptotic protein Bcl-2 (Fig. 5i) clearly demonstrate that nimbolide treatment inhibits pancreatic cancer growth by inducing apoptosis. These findings confirm the *in vitro* observations supporting the therapeutic role of nimbolide in pancreatic cancer.

## Discussion

Despite significant improvements in the technical aspects of cancer detection and management, pancreatic cancer is still thought to be predominantly incurable. The survival of pancreatic cancer patients remains low because of the cancer’s asymptomatic nature, which delays diagnosis at an early stage. Therefore, there is a need to identify pharmacologically safe bioactive molecules that can inhibit pancreatic carcinogenesis without causing any major adverse side effects.

It is well known that plants provide a treasure trove of chemo–therapeutic agents. The present study is the first to investigate whether nimbolide, a limonoid-modified triterpene, can exert anticancer activity against pancreatic cancer. All three pancreatic adenocarcinoma cell lines (HPAC, MIAPaCa-2 and PANC-1) tested were sensitive to the cytotoxic effects of nimbolide at a minimal dose of 3–5 μM. Nimbolide was not highly toxic to normal pancreatic cells (hTERT HPNE) even at higher doses (10 μM). Typical morphological features of apoptosis observed in the nimbolide treated pancreatic cancer cells further confirmed the nimbolide-induced cell death. These findings demonstrate a higher sensitivity of pancreatic cancer cells to nimbolide.

PI3K-AKT signaling is frequently deregulated in many human cancers, which modulates several molecules involved in cell proliferation, growth and metastasis^28^. Nimbolide treatment reduced the activation of AKT in pancreatic cancer cells. Reduced active AKT leads to decreased activity of mTOR and its downstream target, p70s6Kinase, which resulted in the inhibition of cell proliferation. In addition, targeting Ras/Raf/MEK/ERK signaling has also been shown to be critical in enhancing the anti–proliferative and apoptotic effects of therapeutic agents^29^. Consistent with this, we also observed that nimbolide treatment significantly lowered phosphorylated ERK levels in pancreatic cancer cells. These findings demonstrate that nimbolide inhibits pancreatic cancer cell proliferation by altering PI3K-AKT signaling pathway.

The decreased migration, invasion and anchorage-independent growth potential observed upon nimbolide treatment indicates that nimbolide reduces the aggressiveness of pancreatic cancer cells. This further emphasizes that nimbolide can delay and/or inhibit the metastatic potential of pancreatic cancer cells. It is well established that EMT predominantly regulates metastasis and disease progression by regulating tumor invasion, intravasation, extravasation and metastatic dissemination^30^. Nimbolide elicits anti–metastatic effects by blocking EMT, which is evident from its negative effect on pro-EMT markers, such as notch-2, Snail, N-cadherin, Zeb, vimentin and Slug, while in contrast it increased the expression of the epithelial marker E-cadherin^31^.

In cancer cells, ROS has been shown to regulate EMT^32^. ROS plays a dual role in cancer development; excessive production of ROS or disturbances in redox balance leads to a loss of cellular integrity and cell death while a moderate increase in ROS favors cell proliferation^33^,^34^. Several anticancer drugs used to treat various malignant tumors have been shown to induce high levels of ROS^33^. Our results are consistent with previous reports demonstrating that nimbolide induced high levels of ROS generation inhibited pancreatic cancer growth and metastasis.

We used NAC as a ROS quencher, which has been shown to have a plausible route in detoxifying HOCL, OHSCN, -OH, -NO2, CO3, thiyl radicals and removal of H2O2, -O2, and peroxynitrite. Therefore, NAC used in the study quenches different types of ROS generated by nimbolide^35^. Moreover, inhibition of ROS with NAC eliminated the nimbolide-induced cell death, which further confirmed the role of ROS in nimbolide-induced anticancer effect.

ROS plays a vital role in many cellular processes, including autophagy and apoptosis–two major cell death mechanisms^36^. Most chemo–therapeutic agents target apoptosis as a major route to eradicate cancer. Flow cytometric analysis demonstrated the induction of apoptosis in response to nimbolide, as did the elevated expression levels of Bax, cleaved caspase-3, and cleaved PARP and the reduced levels of Bcl-2. We also found the induction of autophagy, as evidenced by the increased localization of GFP-tagged LC3A/B as punctate changes in the cytosol and the elevated expression levels of LC3A/B protein. Induction of autophagy was confirmed by the decreased expression of p62 in nimbolide xenograft tissue. It is well known that increased LCA/B and decreased p62 is the classical sign of autophagy induction^25^,^26^. Understanding the hierarchy between the regulation of ROS and cell death mechanisms (Type I & II) is essential for understanding the molecular mechanisms involved in nimbolide’s anticancer effect. Inhibition of ROS reversed nimbolide-induced apoptosis and autophagy, as evidenced by the decreased expression of cleaved PARP and LC3A/B levels. These data demonstrate that the generation of ROS is an upstream event and is essential for the induction of cell death in nimbolide-treated cells. These findings strongly support the idea that the nimbolide-induced increase in ROS generation may be an effective therapeutic strategy against pancreatic cancer.

Similar to ROS, the role of autophagy in cell death remains as a subject of substantial controversy. Autophagy is well known for its cyto–protective and cyto–toxic effects^37^. Determining the cyto–protective or cyto–toxic role of autophagy is key to understanding the underlying molecular mechanisms of nimbolide-induced cell death. Early- and late-stage autophagy inhibitor studies revealed that nimbolide-induced cell death was not due to autophagy. Treatment of nimbolide causes stress on the pancreatic cancer cells. In response to this these cells induce autophagy as a cytoprotective mechanism. This type of cytoprotective response by pancreatic cancer cells has been reported by several investigators^24^,^38^. We believe that blocking of autophagy with either 3-MA or CQ had minimal effect on cell death since there was not much of autophagy induced in cells without nimbolide. Further, addition of autophagy inhibitors along with nimbolide did decrease cell viability but it was not a major decrease. Moreover, the induction of cell death is mainly through apoptosis as shown by our data.

Our study demonstrates that the increase in autophagy in response to nimbolide is a defensive mechanism by which the pancreatic cancer cells evade death from metabolic stress. In contrast, the inhibition of apoptosis with pan–caspase inhibitor, z-VAD-fmk, effectively blocked nimbolide-induced cell death. Furthermore, the reduced expression levels of cleaved PARP and cleaved Caspase 3 in response to the pan–caspase inhibitor indicate that nimbolide-induced cell death mainly occurs through apoptosis and not via autophagy. Earlier studies have also shown that autophagy promotes pancreatic cancer growth^23^,^39^, whereas apoptosis inhibits pancreatic cancer growth^38^. Numerous reports have described the complex cross-talk between autophagy and apoptosis, but the mechanisms still remain elusive. The molecular interactions between these processes are multifaceted and can cooperate, antagonize or support each other, consequently determining cell fate^37^. To resolve this problem, additional work is required to elucidate the complex interplay between apoptosis and autophagy in determining cell fate. Our data demonstrate the feasibility of activating apoptosis by modulating the redox balance as an effective approach for the development of cancer therapeutics against metastatic cancers.

The *in vivo* xenograft studies demonstrated that nimbolide at low doses significantly inhibited tumor growth without altering the body weights. In an earlier study, nimbolide was reported to have bioavailability in tumor tissue at a concentration of 345 ng/g and 868 ng/g in mice treated with nimbolide at 5 and 20 mg/kg of body weight respectively. Further, HPLC analysis of the plasma samples from the same study detected the bioavailability of nimbolide to be 222 ng/ml and 409 ng/ml in animals treated with nimbolide at a dose of 5 or 20 mg/kg bodyweight respectively^40^. Based on these data it can be suggested that the dose of nimbolide used in our experiment is not only safe, pharmacologically effective and is also highly bioavailable. Our *in vivo* data demonstrates that 5mg/kg body weight dose of nimbolide is highly effective and did not have toxic side effects. Previous literature also provides evidence for pharmacological relevance of these dose levels in clinical translation for effective treatment^12^. Furthermore, nimbolide also effectively inhibited metastasis, which was evident from the finding that no micrometastases were observed in the nimbolide-treated animals. The molecular machineries that were altered in response to nimbolide treatment were similar in both the *in vitro and in vivo* systems. In summary, the *in vivo* data revealed that nimbolide is a safe, natural and effective chemo–therapeutic agent for pancreatic cancer.

In conclusion, our study demonstrates that nimbolide effectively inhibits the proliferation and metastasis of pancreatic cancer cells by increasing ROS generation, which induces mitochondrial-mediated apoptotic cell death. Further investigations in preclinical and clinical settings will potentially lead to the development of nimbolide as an effective therapeutic drug for pancreatic cancer.

## Materials and Methods

This section provides a brief summary of the materials and methods. Detailed information can be found in the Supplementary Information *(SI) Materials and Methods.* All the chemicals, reagents and experimental procedures used were approved by the Texas Tech University Health Sciences Center Institutional Biosafety Committee.

### Cell line culture and reagents

Pancreatic normal (hTERT HPNE) and ductal adenocarcinoma cell lines (HPAC, PANC-1 and MIAPaCa-2) were procured from the American Type Culture Collection (ATCC, Manassas, VA, USA) and maintained at 37 °C in a 5% carbon dioxide incubator with a humidified atmosphere. Dulbecco’s modified Eagle’s media, M3 base Media and RPMI-1640 were procured to culture the above cell lines with similar preparations as mentioned in our prior study^41^. In brief, RPMI-1640 media supplemented with 10% FBS, 100 Units/mL of penicillin, and 100 μg/mL of streptomycin was used to culture PANC-1 and HPAC cells. MIAPaCa-2 was cultured using DMEM with supplemented 10% FBS, 2.5% horse serum, 100 Units/mL of penicillin, and 200 mM glutamine. To maintain hTERT HPNE cells, 75% DMEM and 25% M3 base medium supplemented with 5% FBS, 10 ng/ml human recombinant EGF, 5.5 mM D-glucose and 750 ng/ml puromycin was used.

The 6.5-mm Transwell with 8.0-μm pore polycarbonate membrane inserts was purchased from Corning Incorporated (Corning, NY, USA). BD Matrigel (Bedford, MA, USA) and BD Pharmingen Annexin V-FITC Apoptosis Detection Kit I (San Diego, CA, USA) was obtained from BD Biosciences. Nimbolide was procured from Bio Vision incorporated (Milpitas, CA, USA). BSA as well as N-acetyl-L-cysteine (NAC) and 3-Methyladenine (3-MA) and Chloroquine diphosphate salt (CQ) were purchased from Sigma-Aldrich Corporation (St. Louis, MO, USA). OxiSelect Intracellular Ros Assay kit (Green Fluorescence) was acquired from Cell Biolabs, Inc. Cat: STA-347, (San Diego, CA). MTS reagent [3-(4, 5-dimethylthiazol-2-yl)-5-(3-carboxymethoxyphenyl)-2-(4-sulfophenyl)-2H-tetrazolium] was purchased from Promega (Madison, WI, USA). Mammalian protein extraction reagent (mPER) was acquired from Thermo Scientific (Rockford, IL, USA). Pan-caspase inhibitor (z-VAD-fmk) was procured from Abcam (Cambridge, MA, USA).

Primary antibodies to the following were used in this study: pAKT (sc-101629), AKT (5298), Bcl-2 (sc-783), pERK, (sc-101760) and ERK (sc-94; Santa Cruz, CA, USA); Notch 2 (4530P), Snail (3879), E-cadherin (3195), N-cadherin (4061), Zeb (3396), Vimentin (5741), Slug (9585), Bax (2772), cleaved Caspase-3 (9661), cleaved PARP (9542), pPI3K, PI3K, pPTEN (9549), pmTOR (2974), mTOR (4517), p-p70s6kinase (9206), p70s6kinase (9202), Atg3, Atg5 (D5F5U), Beclin-1 (D40C5), Atg7 (D12B11), LC3A/B (D3U4C), Atg12 (D88H11), and Atg16L1 (D6D5; Cell Signaling Technology, Boston, MA, USA); Sequestosome-1/p62 antibody (ab56416) was procured from Abcam (Cambridge, MA, USA) and β-actin (Sigma Aldrich, (St. Louis, MO, USA). Appropriate secondary antibodies were purchased from Santa Cruz Biotechnology (Santa Cruz, CA, USA).

#### MTS assay

Cell viability was evaluated using the MTS assay as described in our earlier study^41^,^42^, with further details in the Supplementary Information (*SI) Materials and Methods.*

#### Scratch assay

The effect of nimbolide on the migratory capacity of pancreatic cancer cells was measured using the monolayer wound-healing method^41^. Details of the technique are described in the *SI Materials and Methods.*

#### Matrigel invasion assay

The invading ability of pancreatic cancer cells was assessed using a transwell Matrigel invasion assay^42^. The experimental conditions and detailed techniques are described in the *SI Materials and Methods.*

#### Soft agar colony-formation assay

The effect of nimbolide on the clonogenic capabilities of pancreatic cancer cells was tested using the soft agar colony-forming assay^42^. The experimental design and protocol are described in the *SI Materials and Methods.*

#### Immunoblotting

The expression patterns of key signaling molecules were determined using standard immunoblot analyses^41^,^43^. Detailed procedural information is provided in the *SI Materials and Methods.*

#### ROS generation assay

According to the manufacturer’s instructions, intracellular ROS generation was assessed using a ROS assay kit (Oxiselect ROS assay kit). Detailed information is provided in the *SI Materials and Methods.*

#### Immunofluorescence analysis

Pancreatic cancer cells were seeded in 8-well chamber slides at a density of 1 × 10^4^ cells/well and were allowed to grow to 80% confluence. After treatment with nimbolide or NAC alone or a combination of both for 24 h, the cells were fixed with 100% methanol and permeabilized using 0.2% Triton-X in PBS for 20 min. After blocking with 5% BSA, the cells were incubated with the LC3A/B, E-Cadherin, N-Cadherin, Snail and Notch-2 antibodies followed by Alexa fluor 488 (green) or Alexa fluor 568 (Red)-conjugated goat anti- rabbit secondary antibodies (Life Technologies, Grand Island, NY, USA) to detect the activation status of the above mentioned proteins. Images were captured using a Nikon laser scanning confocal microscope^44^.

#### Flow cytometry

HPAC, MIAPaCa-2 and PANC-1 cells were seeded in 6-well plates at a density of 2.5 × 10^5^cells/well and treated with IC_50_ concentration of nimbolide for 24 h. According to the manufacturer’s instructions, the Annexin V-FITC Apoptosis Detection Kit I was used to measure the cell death in pancreatic cancer cells using a FACS Accuri C6 flow cytometer (San Jose, CA, USA).

#### Xenograft model

All *in vivo* experiments were performed in accordance with all guidelines and regulations approved by Texas Tech University Health Sciences CenterAnimal Care and Use Committee. Fifteen 4–6 week old athymic nude mice were obtained from Harlan Laboratories (Madison, WI, USA). The animals were divided into three groups with five animals in each group: control; 5 mg/kg body weight; and 20 mg/kg body weight. All three groups were subcutaneously injected with HPAC cells (3 × 10^6^) in both the right and left flanks. After palpable tumors were observed (~500 mm^3^), nimbolide was administered via intraperitoneal injections to two of the groups, 5 mg/kg body weight and 20 mg/kg body weight^40^, twice weekly, whereas the control group was administered DMSO. These xenograft animal models were monitored continuously for 30 days, and tumor volume and body weight were calculated using the formula 4/3π × r_1_^2^ × r_2_. Control and nimbolide-treated xenograft animal groups were sacrificed at approximately 14 weeks of age. Tumor tissues from all groups were obtained for protein extraction, followed by immunoblotting analysis to study the expression levels of various key signaling protein markers. The excised tumors and major organs such as brain, lung and liver from these animal groups were fixed with 10% formalin and subjected to H&E and histological examination for further studies^45^.

#### Immunohistochemistry (IHC)

The detailed IHC protocol^46^ for key apoptotic, proliferative, EMT and autophagy markers in the tumor xenograft tissues is provided in the *SI Materials and Methods*

#### Statistical analysis

Statistical analysis was performed using Graphpad Prism version 5.03 software (La Jolla, CA, USA). The p-value <0.05 was considered as statistically significant. In brief, the data obtained was analyzed by using repeated measures analysis of variance to assess the dose and time response to nimbolide treatment. Dunnett post-hoc test for multiple comparisons was performed for data with significant differences. In addition, intergroup comparisons were done using paired t-test.

## Additional Information

**How to cite this article**: Subramani, R. *et al*. Nimbolide inhibits pancreatic cancer growth and metastasis through ROS-mediated apoptosis and inhibition of epithelial-to-mesenchymal transition. *Sci. Rep.* **6**, 19819; doi: 10.1038/srep19819 (2016).

## Supplementary Material

Supplementary Information
