# Supplementary material for: Nimbolide inhibits pancreatic cancer growth and metastasis through ROS-mediated apoptosis and inhibition of epithelial-to-mesenchymal transition
Source: Sci Rep. 2016 Jan 25;6:19819. doi: 10.1038/srep19819 (PMC4726267; doi:10.1038/srep19819)
Supplement: Supplementary Information [file srep19819-s1.pdf]

Nimbolide inhibits pancreatic cancer growth and metastasis through ROS-mediated apoptosis and inhibition of epithelial-to-mesenchymal transition

Ramadevi Subramani[a], Ph.D., Elizabeth Gonzalez[b], MS., Arunkumar Arumugam[a], Ph.D., Sushmita Nandy[a], Ph.D., Viviana Gonzalez[c], BS., Joshua Medel[b],MS., Fernando Camacho[b], MS., Andrew Ortega[c], BS., Sandrine Bonkoungou[b], MS., Mahesh Narayan[c] Ph.D., Alok kumar Dwivedi[d], Ph.D., and Rajkumar Lakshmanaswamy[a,b*], Ph.D.

[a] Center of Excellence in Cancer Research, Department of Biomedical Sciences MSB1, Texas Tech University Health Sciences Center, Paul L. Foster School of Medicine, El Paso, Texas-79905.

[b] Graduate School of Biomedical Sciences, Texas Tech University Health Sciences Center, El Paso, Texas-79905.

[c] The University of Texas at El Paso, El Paso, TX 79968

[d] Biostatistics and Epidemiology Consulting Laboratory, Texas Tech University Health Sciences Center, El Paso, Texas-79905.

[*] To whom correspondence should be addressed: Dr. Rajkumar Lakshmanaswamy, Center of Excellence in Cancer Research, Department of Biomedical Sciences, Texas Tech University Health Sciences Center, Paul L. Foster School of Medicine, El Paso, Texas - 79905, USA.

Tel: +1-915-215-4227, Fax: +1-915-783-5222, E-mail: rajkumar.lakshmanaswamy@ttuhsc.edu

Supplementary Information

Supplementary Material and Methods

MTS assay

Cell proliferation was evaluated using the MTS assay as described in a previous study [1, 2]. Normal pancreatic cell line (hTERT HPNE) and pancreatic cancer cell lines (HPAC, MIAPaCa-2 and PANC-1) were seeded in 96-well plates at a density of $0.3X10^4$ cells/well and incubated in a CO_2 incubator overnight. After various treatments for 24 h [nimbolide ranging from 1 to 50 µM, 1 and 5 mM NAC with and without nimbolide (IC_{50} Concentrations viz., 3 µM for MIAPaCa-2, 5 µM for HPAC and PANC-1), autophagy inhibitors (5,10 and 15 µM concentrations of CQ and 0.005 to 2.5 mM concentrations of 3-MA) with and without IC_{50} concentrations of nimbolide, 10 and 50 µM pan-caspase inhibitor (z-VAD-fmk denoted as CI) with and without nimbolide (IC_{50} dose)], MTS was added to each well, and the optical density was measured at 490 nm using a microplate reader after 4 h.

Scratch assay

Cells were seeded on a 6-well plate at a density of 6×10^5 cells/well for HPAC and MIAPaCa-2, and 4.5×10^5 cells/well for PANC-1. After achieving monolayer confluence, a sterile pipette tip was used to create a scratch, and then, the cells were washed twice with PBS. The cells were supplemented with fresh media, treated with nimbolide at their IC_{50} concentrations, and observed for 72 h at 37ºC using the Biostation CT (Nikon Instruments Inc. Melville, NY, USA). Migration data were analyzed using NIS-Element AR software [2].

Matrigel invasion assay

The invading ability of the pancreatic cancer cells was assessed using a total of $5X10^4$ cells in the upper chamber of a transwell polycarbonate membrane coated with 1 mg/ml of Matrigel. Nimbolide was applied using FBS-free media in the appropriate wells for 48 h along with untreated control cells in the upper chamber for an appropriate comparison. Then, 600 µl of growth media with 10% FBS was added as a chemo–attractant in the lower chamber. Cells that invaded the lower chamber were fixed with 0.2% crystal violet in 5% formalin, and then, images from six randomly selected fields were captured using a Nikon Eclipse TS 100 microscope with a magnification of 20X [1]. Experiments were repeated three times.

Soft agar colony-formation assay

Pancreatic cancer cells were seeded on 60-mm dishes with a top layer of 0.7% agar at densities of 2×10^4 cells per dish and a bottom layer of 1% agar. The cells were incubated in medium with or without nimbolide (IC_{50} concentrations). Every 2 days the medium was changed for up to 16 days for MIAPaCa-2, 27 days for PANC-1, and 30 days for HPAC. The cells were incubated at 37ºC and stained with 0.2% crystal violet in 5% formalin solution. Colonies containing 15 cells or more were counted manually, and images were captured using Nikon SMZ 1500 at 10 and 40X magnifications [1].

Immunoblotting

Protein samples were prepared and separated using sodium dodecyl sulfate-polyacrylamide gel electrophoresis and transferred onto polyvinylidene difluoride membranes. After blocking the membranes using 5% bovine serum albumin, blots were incubated overnight at 4ºC with primary antibodies against pAKT, AKT, Bcl-2, pERK, ERK, Notch 2, Snail, E-cadherin, N-cadherin,

Zeb, vimentin, Slug, Bax, cleaved caspase3, cleaved PARP, pPI3K, PI3K, pPTEN, pmTOR, mTOR, p-p70s6kinase, p70s6kinase, Atg3, Atg5, Beclin-1, Atg7, LC3A/B, Atg12, Atg16L1, and β-actin. Primary antibodies were detected with horseradish peroxidase-coupled secondary antibodies and enhanced chemiluminescence [2, 3].

ROS generation assay

Intracellular ROS generation was assessed using the ROS assay kit. A total of $1X10^4$ cells were seeded in a 96-well plate and incubated at 37°C overnight. Following overnight incubation, cells were washed with PBS 3 times and incubated with 2’,7’-dichlorodihydrofluorescin diacetate (DCFH-DA), which is a fluorescent cell compound, at volumes of 1X DCFH-DA in 100 μL/well for 45 min at 37°C. The DCFH-DA solution was removed, and the cells were washed 3 times with PBS. Dose-response analyses were conducted on pancreatic cancer cell lines (HPAC, MIA PaCa-2 and PANC-1) with various concentrations of nimbolide (1, 3, 5, 10, 15, 20 and 25 µM) for 20 min. Similarly, a time-course analysis was performed for 20, 60, 120, 180 and 360 minutes with IC_{50} concentrations of nimbolide respective to each cell line. After the treatments, 3 washes with PBS were performed, and 1x cell lysis buffer was added and mixed thoroughly for 5 min. Then, 150 µL of the mix was transferred to a black cell culture fluorometric 96-well plate for fluorescence measurement. Using a BMG LabTech fluorescence plate reader, the results were read at 480 nm excitation and 530 nm emission. Fluorescence of the samples was also analyzed and captured using a Nikon laser scanning confocal microscope (Nikon Instruments, Melville, NY, USA).

IHC for key apoptotic, proliferative, EMT and autophagy markers in tumor xenograft tissues

Paraffin-embedded tumor tissue sections were used to study the levels and localization of pAKT, E-cadherin, LC3A/B, p62, Bax, and cleaved Caspase 3 using IHC staining. After incubation at 58ºC for 2 h, the slides were deparaffinized and rehydrated in alcohol of serial dilutions oscillating from 100, 95, 70, 50, and 30% ending in a distilled water bath for 5 min. Epitope retrieval was achieved with heating at 95ºC for 15 min with trilogy (Cell Marque, Rocklin, CA, USA). After blocking, the tissues were incubated with their respective primary antibodies and followed by ultra Marque polyscan HRP labeling (Cell Marque, Rocklin, CA, USA). Once stained with chromogen and hematoxylin, the slides were dehydrated with increasing dilutions of ethanol (30, 50, 70, 95, and 100%) ending with a xylene bath. Finally, the sections were sealed with mounting media (Surgipath Medical Industries, Richmond, IL, USA), and the images were obtained using a Nikon Microscope-ECLIPSE 50i at 40X magnification as described [1, 4].

Hematoxylin and eosin Staining

Three- to four-micron sections were cut and placed on a positively charged slide. These embedded sections were deparaffinized and dehydrated gradually followed by H&E staining. H&E-stained pancreatic sections of both control and nimbolide treatment groups were analyzed for metastatic lesions in the brain, lung and liver. Histology images of tumor, brain, lung and liver were captured using a Nikon Microscope- ECLIPSE 50i at 10X magnification.

Supplementary Methods References:

1. Subramani, R. *et al*. Targeting insulin-like growth factor 1 receptor inhibits pancreatic cancer growth and metastasis. *PLoS One* **9**, e97016 (2014).

2. Subramani, R. *et al*. Growth hormone receptor inhibition decreases the growth and metastasis of pancreatic ductal adenocarcinoma. *Exp. Mol. Med.* **46**, e117 (2014).

3. Lopez, R. *et al*. Hyperglycemia enhances the proliferation of non-tumorigenic and malignant mammary epithelial cells through increased leptin/IGF1R signaling and activation of AKT/mTOR. *PLoS One* **8**, e79708 (2013).

4. Arumugam, A. *et al*. Neem leaf extract inhibits mammary carcinogenesis by altering cell proliferation, apoptosis, and angiogenesis. *Cancer. Biol. Ther.* **15**, 26-34 (2014).

Supplementary Figures

Supplementary Fig. S1.

Nimbolide inhibits the metastatic potential of MIAPaCa-2 pancreatic cancer cells

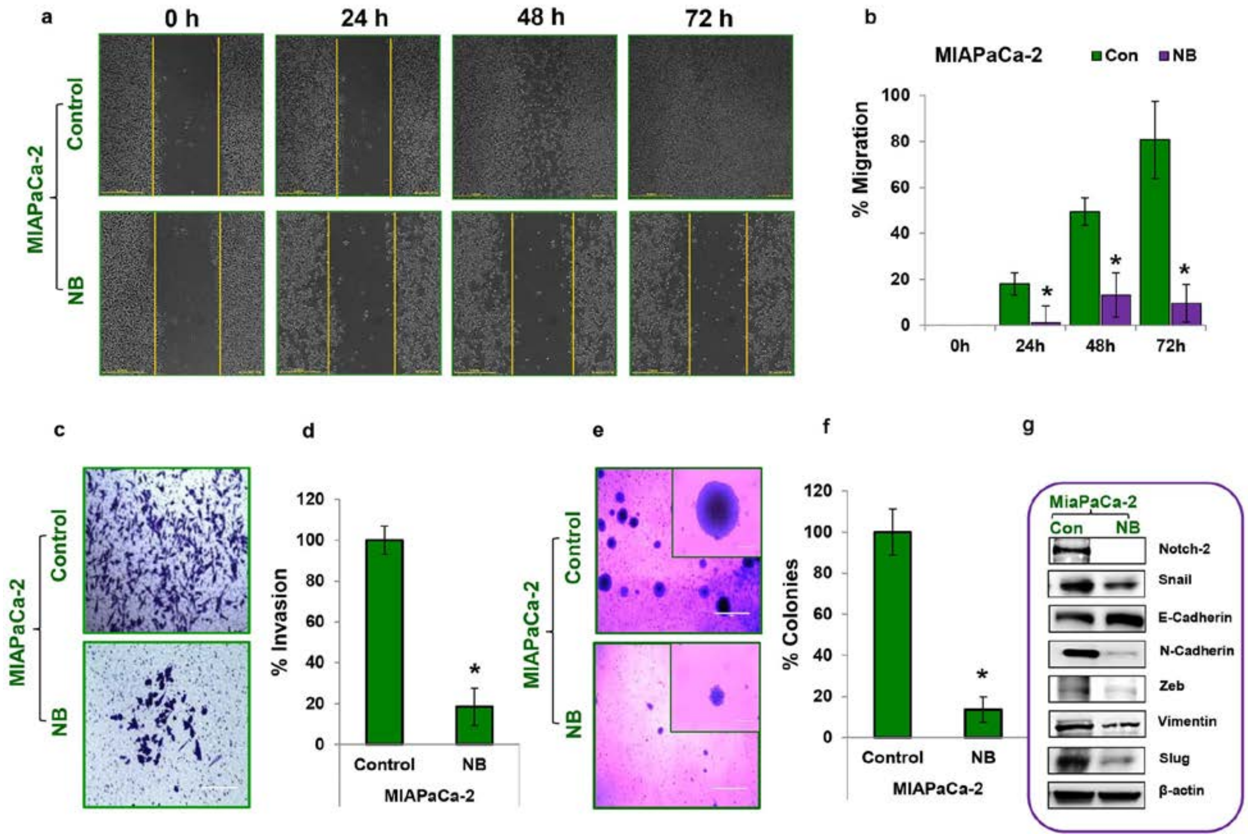

Migration was monitored using a scratch assay using MIAPaCa-2 cells with and without nimbolide treatment for 72 h (a and b). A Matrigel invasion assay was used to determine the invasive capabilities of MIAPaCa-2 cells treated with nimbolide (c and d). A colony-formation assay conducted with MIAPaCa-2 cells cultured with or without nimbolide treatment (e and f). Each bar represents the mean±SEM of three independent experiments, *$p<0.05$. The expression levels of EMT associated proteins with the addition of nimbolide were analyzed by immunoblotting (g). Mann-Whitney U test was performed to determine statistical significance.

Supplementary Fig. S2.

Nimbolide inhibits the migration, invasion, EMT and anchorage-independent growth potential of PANC-1 pancreatic cancer cells

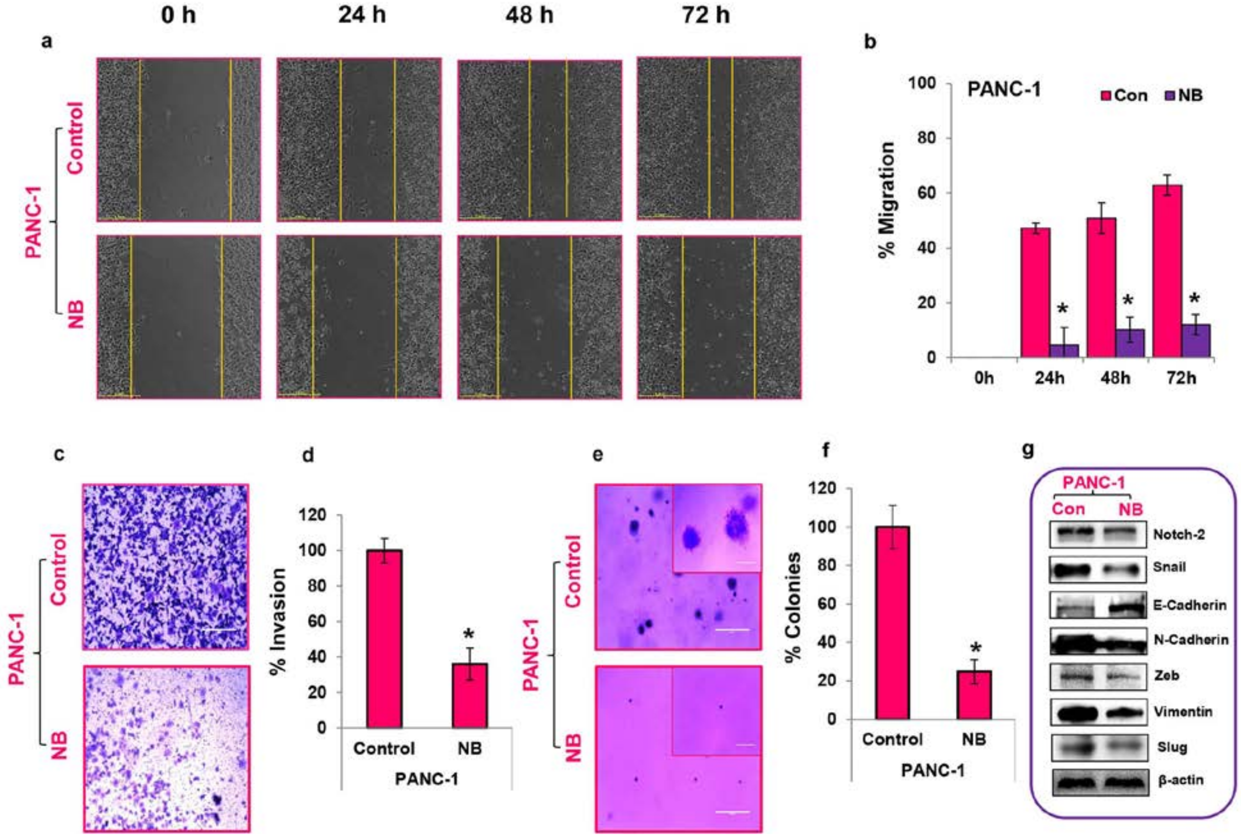

Migration abilities were tested using the scratch assay with untreated control and nimbolide-treated PANC-1 cells (a and b). The invasion potential of PANC-1 cells observed through a Matrigel invasion assay after nimbolide treatment (c and d). The colony-formation assay performed with PANC-1 cells cultured with or without nimbolide treatment to observe anchorage-independent growth potential (e and f). Each bar represents the mean±SEM of three separate experiments, *$p<0.05$. The expression of key EMT markers was observed by immunoblotting analysis after 5 μM nimbolide treatment (g). Mann-Whitney U test was performed to determine statistical significance.

Supplementary Fig. S3.

Induction of ROS, autophagy and apoptosis was recorded in nimbolide-treated MIAPaCa-2 cells

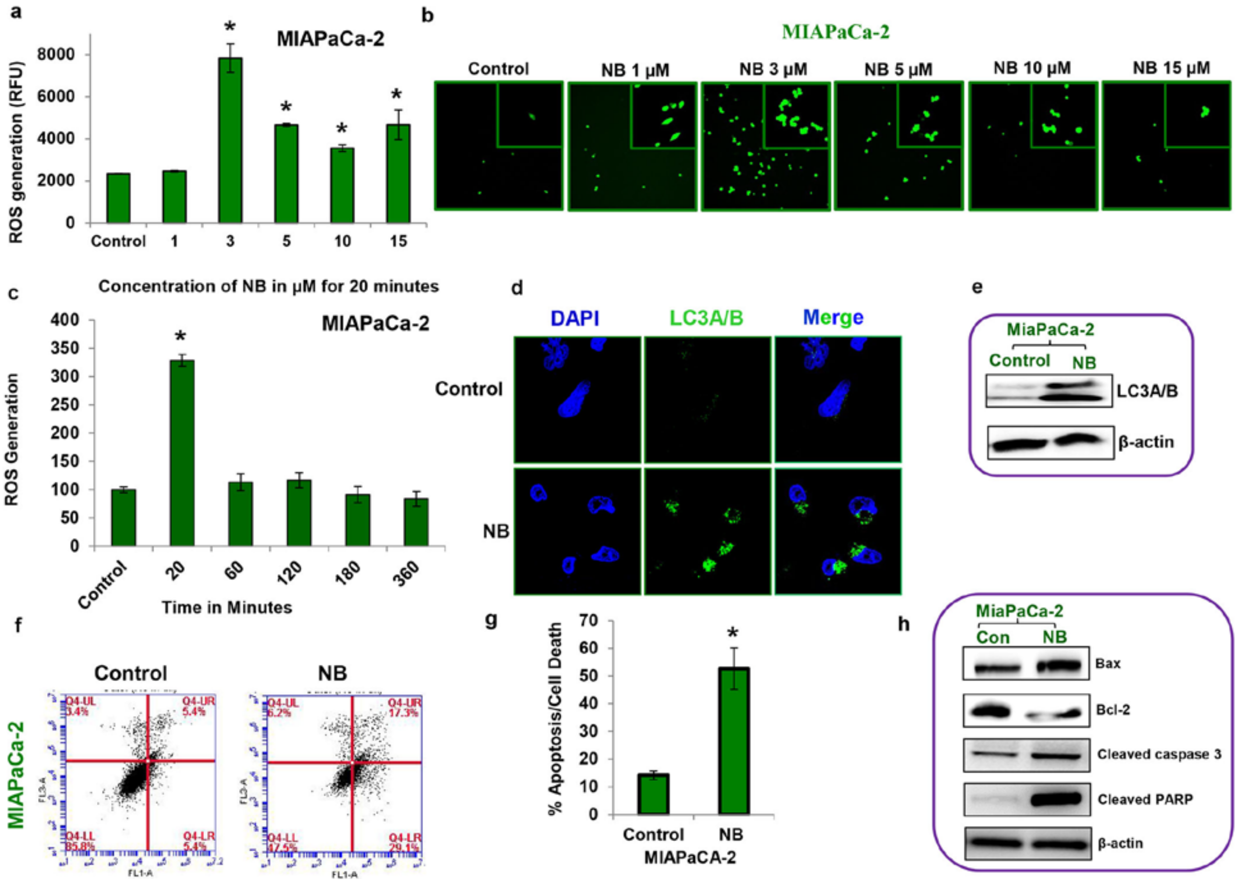

The levels of ROS generation were measured after treatment with nimbolide (1, 3, 5, 10, and 15 μM) in MIAPaCa-2 cells for 20 min using a fluorescence plate reader (a) and fluorescence confocal microscopy (10X and 40X magnifications) (b). Time-course analysis of ROS generation (c). Immunostaining and immunoblot detected the induction of autophagy in nimbolide-treated MIAPaCa-2 cells, which is evident based on the elevated LC3A/B protein expression as punctate lesions in the cytosol (d and e). Increased apoptosis in MIAPaCa-2 cells treated with nimbolide (3 μM) was determined using an Annexin V-FITC Apoptosis Detection Kit I (f and g). The expression levels of Bax, Bcl-2, cleaved caspase 3, and cleaved PARP

confirmed the induction of apoptosis by nimbolide through immunoblotting analysis (h). Each bar represents the mean ± SEM of three separate experiments, *p<0.05. Repeated measures analysis of variance and Dunnett post hoc test was performed to determine statistical significance.

Supplementary Fig. S4.

Nimbolide induces ROS, autophagy and apoptosis in PANC-1 pancreatic cancer cells

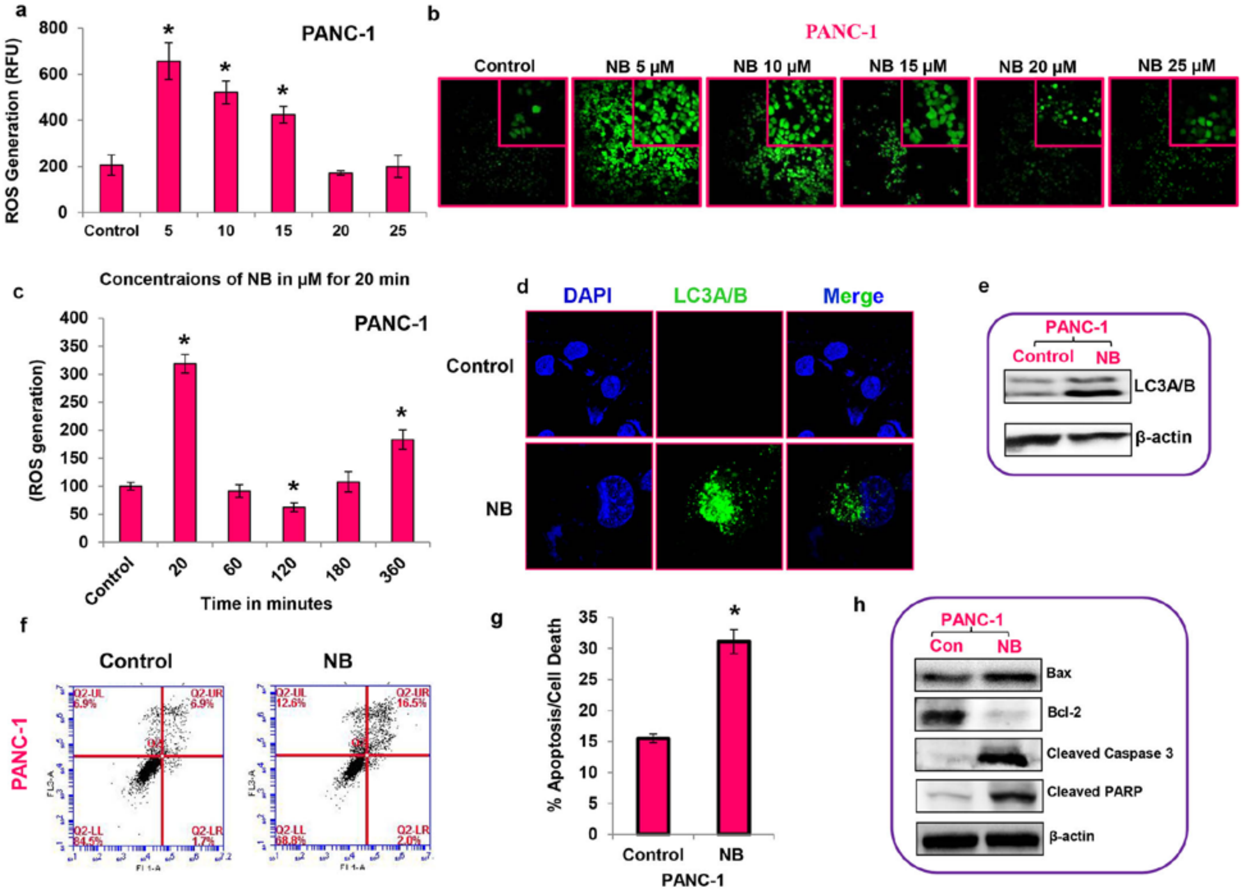

The induction of mitochondrial ROS was measured in PANC-1 cells treated with nimbolide (5, 10, 15, 20 and 25 µM) for 20 min using fluorescence plate reader (a) and fluorescence confocal microscopy (10X and 40X magnifications) (b). Maximum ROS generation through time-course analysis was also evaluated using Oxiselect intracellular ROS assay kit (c). The immunofluorescence of LC3A/B in the nimbolide-treated PANC-1 cells were visualized using

fluorescence confocal microscopy (d), and elevated LC3A/B expression (e) confirmed the induction of nimbolide-induced autophagy. Nimbolide-induced apoptosis was determined using an Annexin V-FITC Apoptosis Detection Kit I (f and g). The protein levels of Bax, Bcl-2, cleaved caspase 3, and cleaved PARP (h). Each bar represents the mean±SEM of three separate experiments, *p<0.05. Repeated measures analysis of variance and Dunnett post hoc test was performed to determine statistical significance.

Supplementary Fig. S5.

ROS regulate both autophagy and apoptosis; ROS induce apoptotic cell death but not through autophagy

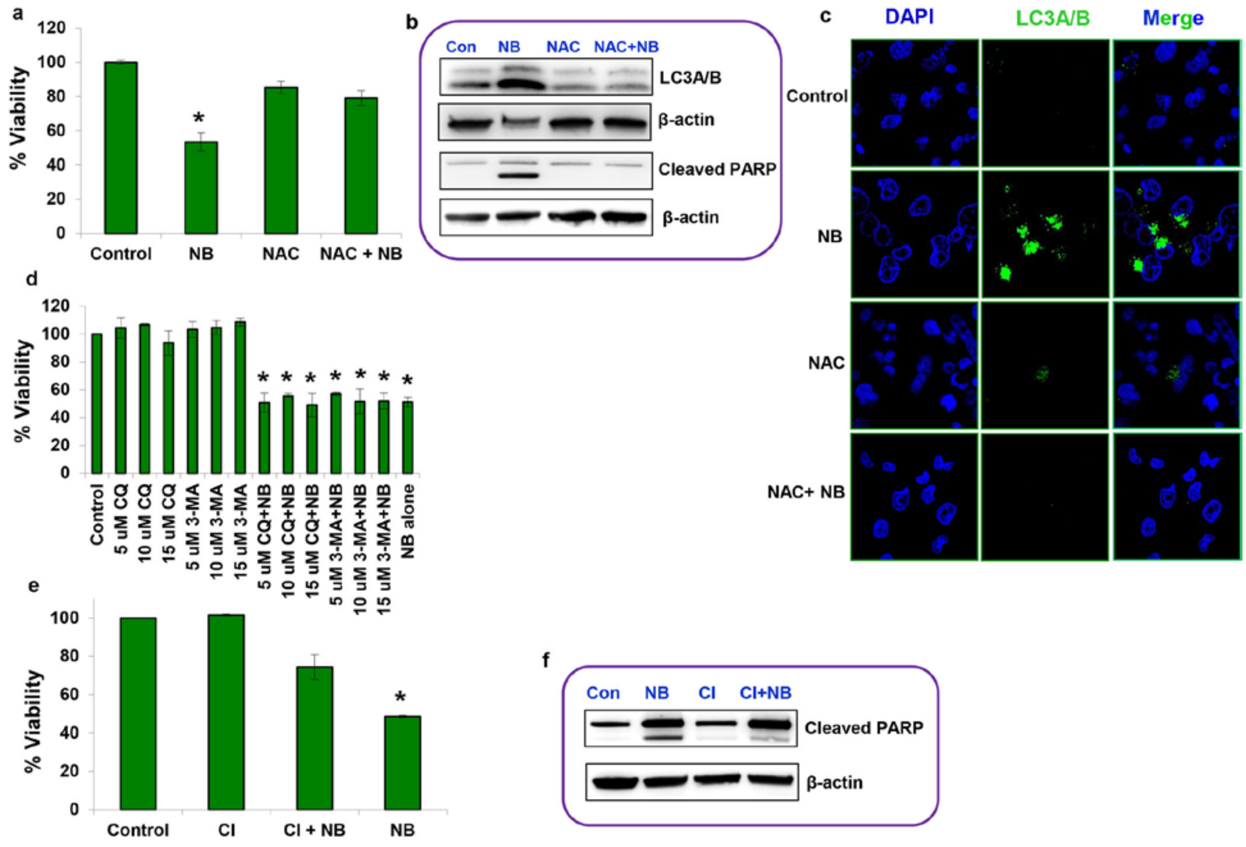

Cell viability of MIAPaCa-2 cells treated with nimbolide (3 μM) or NAC (5 mM) alone or in combination for 24 h (a). The expression levels of LC3A/B and cleaved PARP were evaluated

using immunoblotting (b). Immunofluorescence of LC3A/B detected in the above-mentioned treatment to confirm autophagy regulation via ROS (c). Pretreatment of MIAPaCa-2 cells with CQ and 3-MA followed by nimbolide treatment was used to test cell viability using the MTS assay (d). Cell viability of MIAPaCa-2 cells treated with z-VAD-fmk (10 μM) (denoted as CI) with or without nimbolide (3 μM) (e). The expression levels of cleaved PARP using immunoblotting confirmed that ROS regulated apoptotic cell death (f). Each bar represents the mean±SEM of three separate experiments, *$p<0.05$. Repeated measures analysis of variance and Dunnett post hoc test was performed to determine statistical significance.

Supplementary Fig. S6.

Nimbolide-induced ROS increase pancreatic cancer cell death through apoptosis

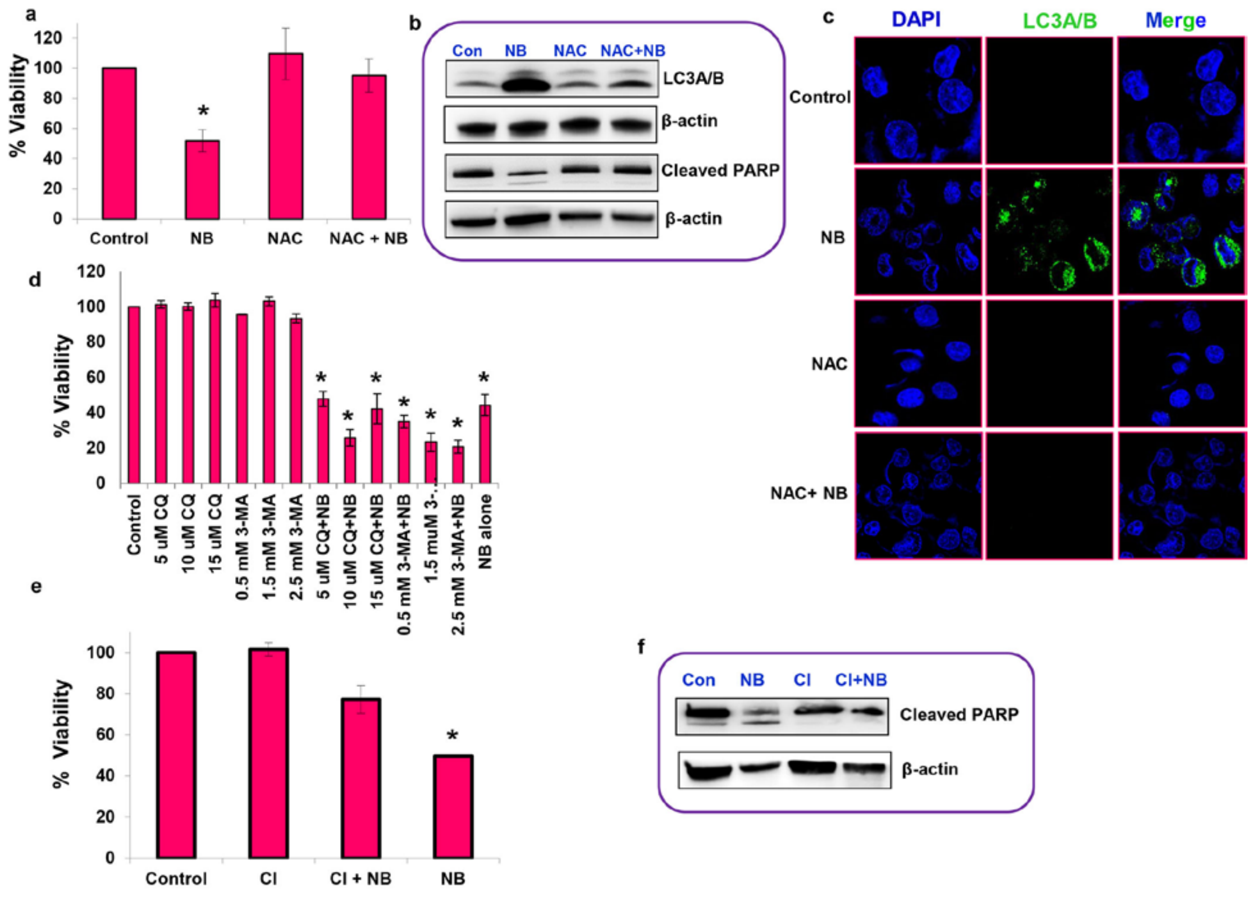

Pre-treatment of NAC (1 mM) followed by nimbolide (5 μM) was used to assess the role of nimbolide-induced ROS on PANC-1 cell viability using the MTS assay at 24 h (a), and the expression levels of LC3A/B and cleaved PARP were evaluated using immunoblotting (b). The expression levels of LC3A/B using immunofluorescence with the above-mentioned treatments were used to assess the regulatory role of ROS on autophagy (c). PANC-1 cells pretreated with CQ and 3-MA in the presence and absence of nimbolide to test the cell viability via MTS assay (d). Cell viability was measured in PANC-1 cells after pretreatment with the z-VAD-fmk caspase inhibitor (10 μM) followed by nimbolide treatment (5 μM) (e). Cleaved PARP expression further confirmed the nimbolide-induced apoptotic cell death (f). Each bar represents the mean±SEM of three separate experiments, *$p<0.05$. Repeated measures analysis of variance and Dunnett post hoc test was performed to determine statistical significance.
